# Anti-Warburg Effect of Melatonin: A Proposed Mechanism to Explain its Inhibition of Multiple Diseases

**DOI:** 10.3390/ijms22020764

**Published:** 2021-01-14

**Authors:** Russel J. Reiter, Ramaswamy Sharma, Sergio Rosales-Corral

**Affiliations:** 1 Department of Cell Systems & Anatomy, Joe R. and Teresa Lozano Long School of Medicine, UT Health San Antonio, San Antonio, TX 78229, USA; sharmar3@uthscsa.edu; 2 Centro de Investigacion Biomedica de Occidente, Instituto Mexicano del Seguro Social, Guadalajara, Jalisco CP45150, Mexico; espiral17@gmail.com

**Keywords:** aerobic glycolysis, mitochondrial melatonin synthesis, hypoxia-inducible factor 1α, pentose phosphate pathway, pyruvate dehydrogenase kinase, pyruvate dehydrogenase complex

## Abstract

Glucose is an essential nutrient for every cell but its metabolic fate depends on cellular phenotype. Normally, the product of cytosolic glycolysis, pyruvate, is transported into mitochondria and irreversibly converted to acetyl coenzyme A by pyruvate dehydrogenase complex (PDC). In some pathological cells, however, pyruvate transport into the mitochondria is blocked due to the inhibition of PDC by pyruvate dehydrogenase kinase. This altered metabolism is referred to as aerobic glycolysis (Warburg effect) and is common in solid tumors and in other pathological cells. Switching from mitochondrial oxidative phosphorylation to aerobic glycolysis provides diseased cells with advantages because of the rapid production of ATP and the activation of pentose phosphate pathway (PPP) which provides nucleotides required for elevated cellular metabolism. Molecules, called glycolytics, inhibit aerobic glycolysis and convert cells to a healthier phenotype. Glycolytics often function by inhibiting hypoxia-inducible factor-1α leading to PDC disinhibition allowing for intramitochondrial conversion of pyruvate into acetyl coenzyme A. Melatonin is a glycolytic which converts diseased cells to the healthier phenotype. Herein we propose that melatonin’s function as a glycolytic explains its actions in inhibiting a variety of diseases. Thus, the common denominator is melatonin’s action in switching the metabolic phenotype of cells.

## 1. Introduction

The glucometabolic reprogramming that occurs in many cancer cells, and in other diseased cells as well [1,2], shifts ATP production away from the mitochondria and into the cytosol; this can occur even when ample oxygen is available and is referred to as aerobic glycolysis or the Warburg effect [3,4]. This change permits cancer cells to rapidly proliferate since it is accompanied by the upregulation of the pentose phosphate pathway (also known as the phosphogluconate pathway or the hexose monophosphate shunt) which provides abundant amounts of ribose-5-phosphate, a necessary constituent that supports nucleotide production [5,6]. Under these conditions, amino acids are also more rapidly converted to proteins [7,8]. These combined actions support the rapid cellular duplication that is characteristic of cancer cells [9,10]. The upregulation of the pentose phosphate pathway also augments the production of pentoses and NADPH [11,12]. The Warburg effect is most often associated with cancer cells [13]. These, however, are by no means the only cells that exhibit this metabolic shift as will be outlined below.

Melatonin is a molecule that has been repeatedly shown to possess oncostatic actions [14,15,16,17,18,19]. As with the Warburg effect, however, melatonin alters the course of many other diseases which, when they develop a pathological phenotype, also resort to engaging in aerobic metabolism [20,21,22].

In this report, we propose that melatonin’s ability to modulate glucose metabolism in pathological cells is a general mechanism by which it impacts the progression of a variety of diseases. This speculation is strongly supported by the recent observations which show that melatonin is probably synthesized in the mitochondria of all healthy cells but likely not in pathological cells [23]. Herein, we also suggest that many pathological cells function as such only during the day and function in a more normal, healthier mode at night, i.e., they only display the pathological phenotype metabolism about half the time.

## 2. Mitochondria, An Ecosystem in Which Melatonin is Produced and Functions

Mitochondria are abundant in all cells (a few exceptions) and their number varies according to the energetic requirements of the cell in which they reside. Thus, predictably, cardiomyocytes would be expected to have more numerous mitochondria than some other cells, which they do [24].

In normally functioning aerobic cells an estimated 90–95% of total ATP production occurs in the mitochondria [25]. This percentage may be reversed when cells adopt a Warburg-type metabolism in which a large percentage of the ATP is manufactured in the cytosol. The total amount of ATP produced in an average adult human is estimated to be 40 kg/day [26].

Under optimal normal conditions [27], a small percentage of the oxygen is reduced by either one, two, or three electrons thereby leading to the generation of the superoxide anion radical (O_2_•−) which is readily enzymatically dismutated to hydrogen peroxide (H_2_O_2_) or it couples with nitric oxide (NO•) to form the peroxynitrite anion (ONOO^−^) [28,29,30,31]. After its formation, H_2_O_2_ is either enzymatically removed by catalase and glutathione peroxidase [32,33], or in the worst-case scenario, it forms the hydroxyl radical (•OH) as a result of the Haber–Weiss reaction [34]. Both ONOO^−^ and •OH are highly reactive and account for a large majority of nitro/oxidative stress that mitochondria sustain [30,31,35,36].

Melatonin is well documented as to its ability to prevent nitro/oxidative stress under varied experimental conditions where it has been tested [37,38,39,40,41,42,43,44]. This high efficiency relative to other antioxidants [45,46,47,48] presumably stems from its direct ROS/RNS scavenging activities [49,50,51,52,53,54,55,56] and, perhaps more so, from its facility to stimulate antioxidative enzymes, to inhibit pro-oxidant enzymes, to restore the levels of other radical scavenging agents such as glutathione [57,58,59,60], and to synergistically interact with classical radical scavengers in protecting against oxidative stress [45,46].

Many of the studies cited above confirm the actions of melatonin at the mitochondrial level. When measured in hepatic and neural subcellular factions, melatonin was found to be much higher in this organelle compared to other components [61], which is certainly consistent with its actions at this site and would be highly fortuitous considering the lofty ROS generation in this organelle [62,63,64]. To prove that mitochondrial melatonin was not of pineal origin, these values were also estimated in the mitochondria of long-term pinealectomized animals and no decrement was observed, affirming that pineal-derived circulating melatonin was not responsible for the mitochondrial levels of melatonin in these organs. Since blood melatonin levels are exclusively pineal-derived, the observations of Venegas and colleagues [61] strongly inferred that this melatonin was a synthetic product of these cells, in this case, neurons/glia and hepatocytes. Armed with these data and the earlier findings of Manchester et al. [65] which suggested that melatonin may be synthesized in ancient prokaryotic bacteria which are believed to be the precursors of mitochondria in eukaryotic cells [66,67], we subsequently speculated that mitochondria of all cells may produce melatonin for their own use [68] and do not release it into the systemic circulation. Thus, there are two pools of melatonin in vertebrates [69,70], a releasable pool derived from the pineal gland and a non-releasable pool from the mitochondria of all other cells. Because of the two sources of melatonin, Zhao et al. [70] estimated that less than 5% of the total melatonin vertebrates synthesize is actually derived from the pineal gland.

More than three decades before Venegas and colleagues [61] identified the high levels of immunoreactive melatonin in the hepatic and brain cell mitochondria collected from animals deprived of what is the exclusive source of circulating melatonin; that is, the pineal gland, the first data suggesting the mitochondria may be the intracellular site of melatonin synthesis was published. In the mid-1970s, Kerenyi et al. [71] attempted to identify the location of the rate limiting enzyme in melatonin synthesis, N-acetyltransferase (NAT), in rat pinealocytes using an immunocytochemical method (copper ferrocyanide method) available at the time. What they observed is that the immunoreactive product for NAT was only found in the pineal cell mitochondria, indicating this organelle is likely involved with melatonin synthesis. These findings were not given much credibility, however, since the pineal glands were collected from animals during the light period, when the biochemical evidence had shown that this enzyme is almost absent during the day [72]. Further, the study only examined pineal tissue where melatonin synthesis was known to occur [73,74].

Following the discovery of melatonin in non-pineal tissue mitochondria [61] and the explanation provided for these organelles being a likely site of melatonin production in cells [75], finally in 2016, the first serious attempt was made to determine where in non-pineal cells melatonin is synthesized. He and coworkers [76] purified mitochondria from superovulated mouse oocytes on which to test their presumption. Only when the mitochondria were incubated with serotonin was melatonin formed and released into the incubation medium; this proved that this organelle is indeed capable of converting serotonin to N-acetylserotonin and the latter molecule to melatonin; this pathway requires the enzymes NAT and acetylserotonin methyltransferase (ASMT) [77] which are obviously both present in the oocyte mitochondria. Perhaps the most important aspect of this study, however, was that they used mitochondria isolated from oocytes. These maternally-derived organelles are the forerunners of all cellular mitochondria in adult mammals. Thus, since they produce melatonin it could be reasonably speculated that this ability is retained by all subsequent mitochondria derived from these organelles [64].

The most convincing data related to the ability of mitochondria to produce melatonin comes from a publication by Suofu et al. [78]. They isolated non-synaptosomal mitochondria from the brain of mice and identified the presence of both AANAT and ASMT proteins as well as the chaperone 14-3-3-ζ; the positive control was pineal mitochondria where the enzyme proteins were also obviously present. Furthermore, they tested whether there was a day:night difference in AANAT protein in the organelles of both tissues and found a rhythm in the pineal but not in neural mitochondria.

Suofu and coworkers [78] also performed a series of studies in which AANAT was knocked out with the mitochondria then being exposed to an oxidative challenge; this showed that in the absence of melatonin there was a large increase in oxidative stress, proving that the methoxyindole functions as an antioxidant in mitochondria, an organelle known to generate large amounts of ROS. Finally, Suofu et al. [78] incubated purified mitochondria with deuterated serotonin and observed they produced deuterated melatonin and related indoles. These studies, more than any others, provide compelling data for the mitochondrial synthesis in non-pineal cell mitochondria; this is consistent with the high levels of melatonin measured in hepatocyte and neural mitochondria as reported by Venegas et al. [61] and with the speculation that all cells, which contain mitochondria produce melatonin. They also document there are two pools of melatonin, one releases it into the circulation, and one which does not [23,68].

In 1987, we speculated that melatonin could be rapidly taken up by tissues from the blood when it was observed that high nocturnally elevated melatonin levels dropped precipitously in stressed rats despite its continued high synthesis in the pineal gland [79]. The likely mechanisms for melatonin uptake by peripheral cells have only recently been better defined, however. The results of Hevia et al. [80], as also summarized by Mayo et al. [81], suggests that rather than simple diffusion of highly-lipid soluble melatonin through cell membranes, its uptake is an active process that involves the glucose transporter (GLUT). In this process, melatonin may compete with glucose for entrance into cells, a finding that could have significant bearing on the role melatonin has in influencing glucose uptake by cancer cells and in diabetic conditions [80,82] and also in terms of intracellular ROS management. More recently, another route of melatonin transport into cells has been described. A study using five cancer cell lines provided compelling evidence that the human oligopeptide transporters PEPT 1/2 are instrumental in mediating the cellular uptake and, for the first time, the mitochondria uptake of melatonin where it would have immediate access to short-lived reactants produced during oxidative phosphorylation [OXPHOS]. To date, the PEPT 1/2 transporters as they relate to melatonin uptake have only been described in the five cancer cell lines utilized by Huo and colleagues [83]. It is presumed they exist in normal cells as well where they function similarly. These observations provide credence for the data showing that melatonin is targeted to mitochondria [62] and its ability to diminish ROS immunofluorescence in these organelles [38,48].

**Figure 1:**
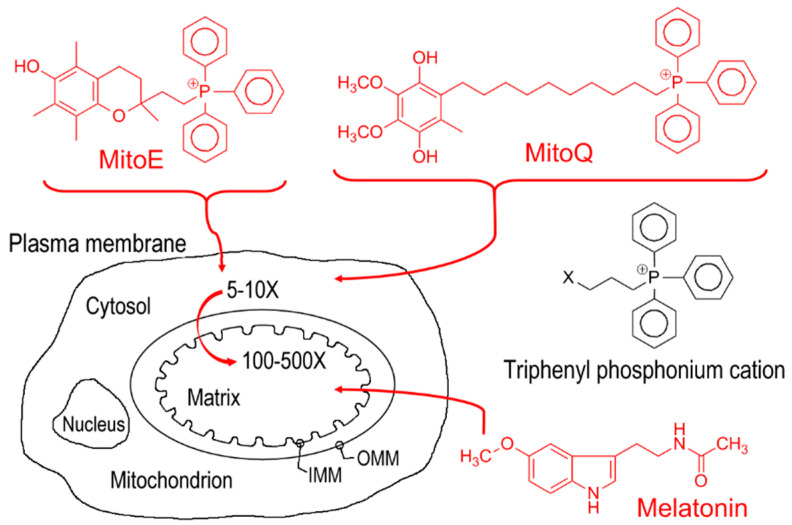
Coupling native vitamin E and coenzyme Q10 to the triphenyl phosphonium cation (at position X) creates molecules referred to as Mito E and Mito Q. These synthetically produced antioxidants are more lipid-soluble than their native precursors and, as a result, they concentrate in the mitochondria, the site of maximal reactive oxygen species generation, up to 500-fold. When these modified antioxidants were compared with melatonin at equimolar concentrations, in terms of their anti-inflammatory and antioxidant actions in mice treated with the bacterial toxins lipopolysaccharide and peptidoglycan G, melatonin was equally effective on most parameters measured and in some cases, melatonin was superior. This attests to the outstanding efficacy of melatonin as to its function as an anti-inflammatory agent and use as an antioxidant.

In consideration of the ability of melatonin to be transported into mitochondria and its efficacy in neutralizing ROS in these organelles [38,39,48], a question arises as to the effectiveness of melatonin to accomplish this relative to better known antioxidants. There is only one publication where this issue was adequately addressed in vivo [39]. Earlier it was mentioned that the pharmaceutical industry has an abiding interest in molecules targeted to mitochondria [36]. To boost the ability of vitamin E and coenzyme Q10 to enter the mitochondria, these molecules were chemically modified to increase their lipid solubility which would enhance their uptake by this organelle. The resulting products, referred to as Mito E and Mito Q, respectively, were reported to concentrate in the mitochondria up to 500-fold relative to the unaltered versions of these agents (Figure 1). When these “super” antioxidants were compared with melatonin at equimolar concentrations, they were not better than native melatonin and for some indices, they were less effective than melatonin in reducing the inflammatory and pro-oxidative actions mediated by the administration of highly toxic bacterial endotoxins. The relevance of these findings extend beyond the anti-inflammatory and antioxidant properties of melatonin inasmuch as mitochondrial metabolism is commonly disturbed in cancer cells, e.g., the Warburg effect, which melatonin has been shown to reverse [69,84,85].

**Figure 2:**
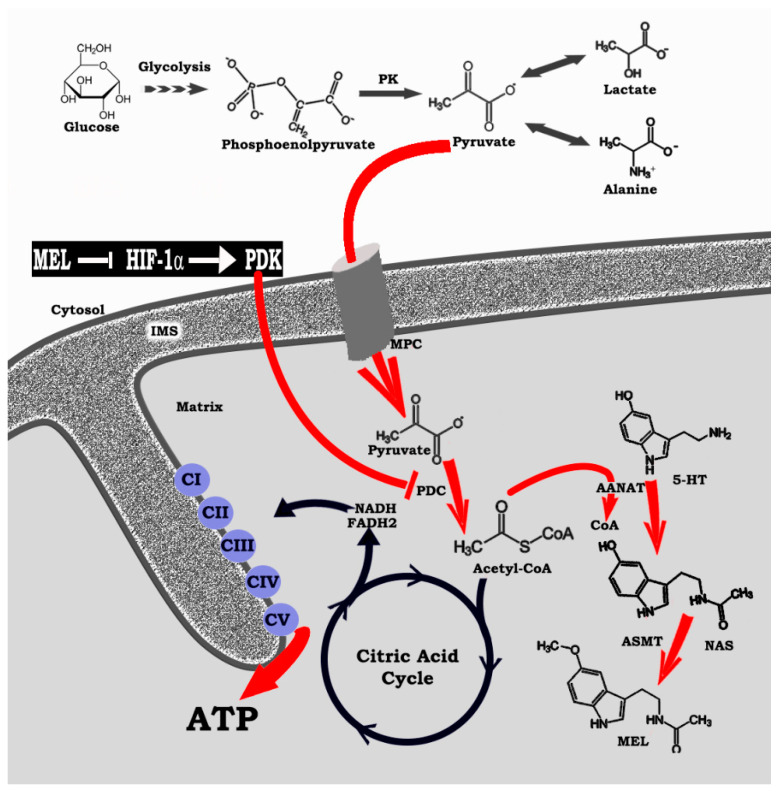
The association of intramitochondrial pyruvate metabolism and its relation to locally-produced melatonin is illustrated here. Only when pyruvate is enzymatically converted to acetyl coenzyme A (acetyl-CoA) by pyruvate dehydrogenase complex (PDC) is melatonin synthesized in these organelles. In healthy cells the glucose metabolite, pyruvate, is transported across mitochondrial membranes by membrane pyruvate carrier (MPC) into the matrix. Here, pyruvate is acted upon by PDC to generate acetyl-CoA, a critically important factor for feeding the citric acid cycle (tricarboxylic acid cycle; Krebs cycle) and aiding the respiratory chain complexes in the production of ATP while also reducing reactive oxygen species production. In addition, acetyl-CoA serves another important task in the mitochondrial matrix by serving as a co-factor/co-substrate for the rate limiting enzyme in melatonin production arylalkyl-N-acetyltransferase (AANAT), which converts serotonin (5-HT) to N-acetylserotonin (NAS). Once formed, N-acetylserotonin forms melatonin (N-acetyl-5-methoxy-tryptamine) under the influence of acetylserotonin methyltransferase (ASMT). Locally-formed melatonin serves a variety of functions in the mitochondrial matrix by stimulating SIRT3, which upregulates a variety of essential actions. In diseased cells, pyruvate is excluded from the mitochondria since PDC is strongly downregulated by pyruvate dehydrogenase kinase (PDK) which is stimulated by hypoxia inducible factor 1-α Without the synthesis of acetyl-CoA in the matrix of diseased cells, efficient ATP production is compromised and melatonin synthesis is precluded. Thus, pyruvate is retained in the cytosol where it is converted by the action of lactate dehydrogenase to lactate. Circulating pineal-derived melatonin can, however, enter cells (both healthy and diseased cells) to inhibit HIF-1α leading to the down regulation of PDK, the disinhibition of PDC and the production of acetyl-CoA which allows melatonin to be locally produced. Endogenous blood melatonin concentrations are only elevated during the night in young and middle-aged individuals. Thus, glucose metabolism is different during the day and night in diseased cells.

Melatonin is very much “at home” in mitochondria where it is both synthesized [76,78] and taken up from the circulation [81,83,86] (Figure 2). This location provides melatonin with significant advantages in terms of its ability to control cancer cell metabolism as well as relieving cells, when necessary, of undo oxidative stress. Its ability to both modulate glucose uptake and its intracellular metabolism also makes it an important agent in resisting cancer since these cells typically have a “sweet tooth” and require excessive amounts of glucose to sustain their growth [87,88]. Having identified these actions, however, the reader may be left with the impression that these functions are universal and unwavering under all metabolic circumstances. This, however, is not the case; as with some other molecules [89,90] melatonin’s actions are context specific [91,92]. An adequate explanation for this plasticity is not currently available.

## 3. The Warburg Effect: A Focal Point for a Number of Diseases

**Figure 3:**
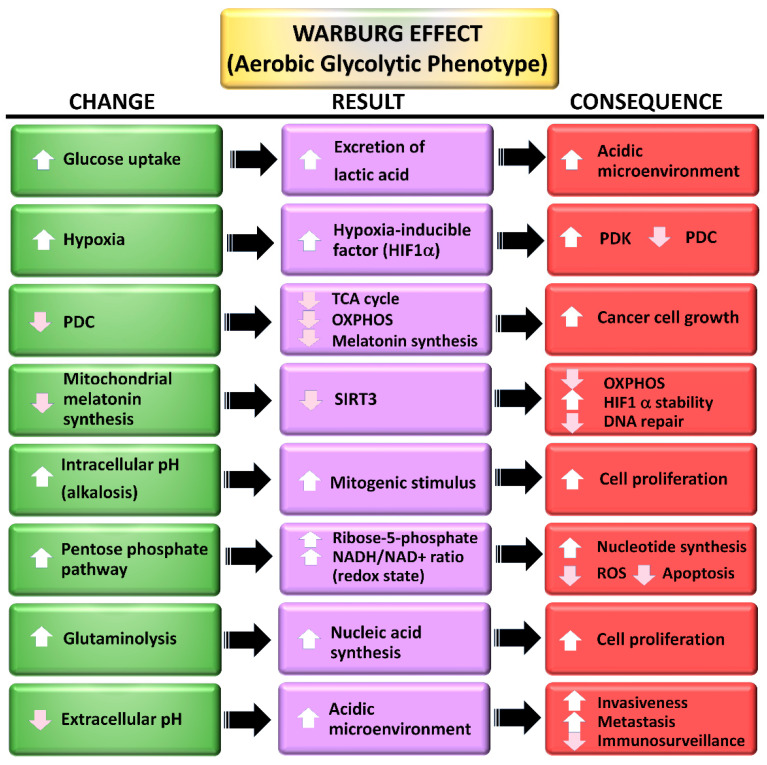
The Warburg effect which has been most thoroughly studied in solid tumor cells, is not unique to cancers as indicated in the current review. The major changes include and upregulated glucose metabolism pathway which is associated with a large number of other alterations as summarized in this figure. Another major alteration that occurs is that the cells reduce the activity of the tricarboxylic cycle (TAC) and oxidation phosphorylation (OXPHOS) for ATP production by precluding the synthesis of acetyl CoA due to the down regulation of the associated enzyme pyruvate dehydrogenase complex (PDC). This shunts pyruvate into the cytosolic fermentation pathway which results in the production of lactate. The pentose phosphate pathway (see Figure 4) is also activated leading to the synthesis of a variety of molecules that ensure nucleotide synthesis and underpin cellular proliferation. HIF-1α = hypoxia inducible factor-1α; PDK = pyruvate dehydrogenase kinase; SIRT3 = sirtuin 3.

For decades, the Warburg effect (aerobic glycolysis) was thought to be unique to cancer cells (Figure 3). It does not, however, occur in all cancer cells and it often develops under other pathological conditions as well (see below). The bulk of the information related to the molecular events associated with aerobic glycolysis obviously came from studies of cancer cells; mechanistically, it is generally assumed that the processes are similar in all pathological cells where it occurs.

One major molecular disruption involves PI3K/AKT, the signaling processes of which are often perturbed [93,94,95]. These changes advance the molecular biological events that enhance glucose metabolism and lead to increased cellular proliferation and cellular invasion of adjacent tissues allowing eventually for metastasis. Further, AKT promotes intracellular events including the expression and activity of glucose transporters (GLUTs) in the cell membrane [96,97,98]. AKT, due to its activation of mTOR, is important for enhancing lipid biosynthesis and triggering the rapid transport of glucose into cancer cells which hastens glycolysis. Moreover, mTOR stimulates hypoxia-inducible 1α (HIF-1α), a major player in indirectly inhibiting mitochondrial oxidative phosphorylation and fueling aerobic glycolysis [99,100]. The oncogene cMyc also is involved with the regulation of glycolysis due to its enhancement of the expression of GLUT genes and stimulation of pyruvate dehydrogenase kinase (PDK) and lactate dehydrogenase A (LDHA), the latter metabolizing pyruvate to lactate. Lactate is then quickly released from the cell and contributes to the acidification of the extracellular microenvironment, a situation also beneficial to cellular invasion and metastasis [101,102,103].

Activation of HIF-1α is an adaptative response coupled to intracellular hypoxia resulting from the depressed oxygen supply that develops when rapidly dividing neoplastic cells exceed the ability of neovasculogenesis to keep pace with the growing solid tumor. Within a given solid tumor there may be a gradient from near the center of the tumor where the oxygen tension is lowest to the periphery where the cells may be normoxic; this is also reflected in the intensity of HIF-1α expression [104,105,106,107]. Unequivocally, HIF-1α has a central role in orchestrating the processes that allow cancer cells (and other diseased cells) to accommodate aerobic glycolysis [108,109]. Under normoxic conditions, in which most cells exist, HIF-1α subunits are ubiquitinated by the von Hippel–Landau complex and quickly undergo proteasomal degradation, so it has little effect on glucose metabolism or oxidation [110,111]. Under hypoxic conditions, HIF-1α is not polyubiquitinated so it is not degraded by the proteosome; thus, it becomes available for manipulating glucose metabolism.

One critical function of HIF-1α is the stimulation of the mitochondrial PDK. PDK is the enzyme that serves as the gatekeeper for the entrance of pyruvate, the end product of glycolysis, into the mitochondria [112,113]. The activation of PDK, by HIF-1α, or any other means, leads to the phosphorylation of the E1α subunit of pyruvate dehydrogenase complex (PDC), which inactivates it [114,115]. When this link is broken due to the inhibition of PDC, pyruvate is re-routed to the cytosol where it undergoes fermentation to lactate. The processes, collectively identified as the Warburg effect (Figure 3), are commonly associated with solid tumors and contribute significantly to their hardiness, invasiveness and their metastatic capability as well as rendering them resistant to radio- and chemotherapies [116,117]. Thus, drugs that disinhibit PDC by maintaining it in an unphosphorylated configuration and catalytically active state, are considered potential anticancer agents [118,119].

**Figure 4:**
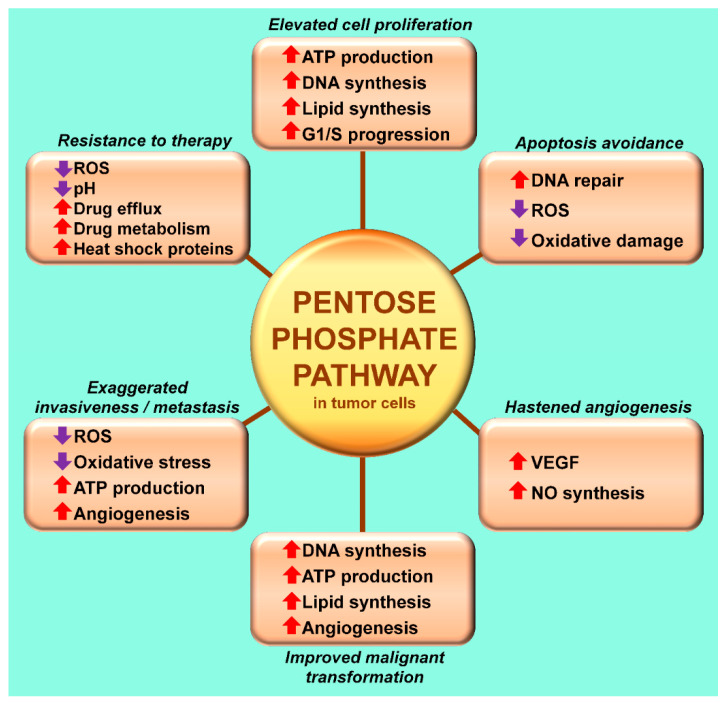
The pentose phosphate pathway becomes highly activated during aerobic glycolysis (the Warburg effect) (Figure 3) when pyruvate dehydrogenase kinase inhibits pyruvate dehydrogenase complex. This pathway, the initial molecule of which is glucose-6-phosphate, generates the necessary molecular building blocks for the synthesis of nucleotides, which supports cell proliferation, invasiveness and metastasis of cancer cells. Herein, the authors propose that inhibiting the Warburg effect, which interferes with processes that accelerate cell proliferation, etc., may be a common mechanism for melatonin to modulate disease progression not only in cancer but also in other diseased cells that utilize aerobic glycolysis. G1/S = growth 1 to synthesis; ROS = reactive oxygen species; VEGF—vascular endothelial growth factor.

Derailing pyruvate metabolism away from the mitochondria and into the cytosol is associated with other metabolic shifts. With the absence of acetyl-CoA, which normally feeds the TCA cycle and supports OXPHOS, due to inactivation of PDC by PDK, mitochondrial ATP production is also often compromised. This reduction is compensated by the accelerated cytosolic conversion of ADP to ATP that accompanies the elevated glycolysis [120,121]. Additionally, to support the high synthetic requirements of cells that adopt the Warburg-type metabolism, they employ the pentose phosphate pathway (PPP) [122] (Figure 4). This pathway is initiated when glucose-6-phosphate is enzymatically converted to glucose-1,5-lactone 6P by the glucose-6-phosphate dehydrogenase; this is the rate limiting step in the PPP and the pathway yields 2 mol NADPH and CO_2_.

Before the mitochondrial synthesis of melatonin was identified, a presumably unexpected phenomenon was reported by Blask and colleagues [84] related to cancer cell glucose oxidation for which there was no known explanation. When glucose uptake and lactate release was estimated in venous blood drained from MCF-7 human mammary cancer cells growing in immunocompromised rats, the workers found that during the day glucose uptake and lactate secretion were highly elevated. In the tumors collected at the same time, H^3^-labeled DNA synthesis and total DNA were also both elevated. These measurements indicated that during the day the cancers were using Warburg-type metabolism and were actively synthesizing DNA to support rapid cell division. Remarkably, at night these measures were drastically different. Thus, glucose uptake and lactate secretion were low as were the DNA parameters. Clearly the tumors were not using Warburg-type metabolism at night, but seemingly had reverted back to conventional mitochondrial glycose oxidation. This marked day: night metabolic rhythm [85] was driven by the circadian variation in blood melatonin concentrations since it only occurred when melatonin in the blood displayed the typical high levels at night and low values during the day. When nighttime melatonin concentrations were depressed by the exposure of the tumor-bearing rats to light at night, the cancers exhibited the Warburg-type metabolism both during the day and also during the subjective night, that is, they were functioning as cancer cells throughout the 24-h period. This was supported by the observation that the mammary tumors in the melatonin suppressed rats grew faster than those in the animals that had a conventional day: night melatonin rhythm.

In a study by Mao et al. [123] results similar to those of Blask and coworkers [84] were reported except the cancer type xenografts studied were human leiomyomas, a malignant tumor type with high metabolism. In this study, orally administered melatonin, creating what the authors referred to as low pharmacological concentrations, reversed the Warburg effect and concurrently reduced tumor growth and metastasis, changes that were expected to accompany a reduction of glucose oxidation in the mitochondria. The results also provided essential information related to the signaling pathways by which melatonin operates. Thus, when cancer cells were treated with both melatonin and S20928, a non-selective melatonin receptor (MT1 and MT2) blocker, aerobic glycolysis was not reversed; this is the first evidence that melatonin determines the intracellular route of pyruvate metabolism in cancer cells is receptor-mediated.

An important contribution to this issue was also made by Sanchez-Sanchez et al. [124] who compared the dissimilar oxidative response to melatonin in two contrasting tumor cell types; that is, Ewing sarcoma cells in which melatonin is cytotoxic and in chondrosarcoma cells where melatonin inhibits cell proliferation but does not kill the cells. As indicated by the elevated glucose uptake, increased lactate dehydrogenase activity and the stimulation of HIF-1α, only the Ewing sarcoma cells displayed aerobic glycolysis and, as in the previous report [124], melatonin reversed these changes and prevented the cytotoxicity of melatonin. The evidence supports the idea that melatonin is only cytotoxic to cancer cells that develop a Warburg-type metabolism and it explains the differential responses of Ewing sarcoma and chondrosarcoma cells. Neither Mao and colleagues [123] nor Sanchez-Sanchez et al. [124] actually examined the potential changes in the PDK/PDC axis which determines whether tumor cells utilize aerobic glycolysis or OXPHOS as a basis of energy production. A key factor in the regulation of the PDK/PDC axis is the activity state of HIF-1α; Sanchez-Sanchez et al. [124] did report the HIF-1α was upregulated in Ewing sarcoma cells before, but not after the application of melatonin.

After examining the findings of the three previously discussed reports [84,123,124], in 2019 we pointed out the essential role of acetyl-CoA for the synthesis of melatonin in the mitochondria matrix [125]. At that time, it was known that melatonin has multiple intramitochondrial functions [23,37,46,48,64], that it is in high concentrations in this organelle [61] and that it has the enzymatic network for melatonin synthesis to proceed [76,78]. We realized for this to occur; however, the mitochondria required a source of acetyl-CoA since it is a necessary co-factor/co-substrate for the rate limiting enzyme in melatonin synthesis, AANAT. Thus, in addition to coupling with oxaloacetate to feed the TCA cycle and maintain OXPHOS [126] at an optimal level, acetyl-CoA must also be present to preserve the intramitochondrial concentrations of melatonin (Figure 2). This becomes of critical importance when cells assume a Warburg-type metabolism. In this situation, the enzyme that irreversibly converts pyruvate to acetyl-CoA, PDC, is downregulated which causes a depletion of acetyl-CoA and, in turn, the deprivation of melatonin. This loss is highly consequential in terms of mitochondrial physiology. As one example, since locally-produced melatonin drives SIRT3 activity which deacetylates and activates superoxide dismutase (SOD2) [127,128], a critical antioxidant enzyme, the molecular framework of the mitochondria is highly vulnerable to oxidative stress since the damaging ROS are not removed. This drop only occurs in diseased cells since they most often utilize Warburg-type metabolism.

Melatonin exerts a marked regulatory effect on the PDK/PDC axis due to its ability to directly or indirectly inhibit HIF-1α [69,124,129]; as a result, it determines the pyruvate metabolic profile of those cancer cells that have been investigated and perhaps other diseased cells as well [130].

## 4. HIF-1α, a Regulator of Regulators: Influence on Mitochondrial Melatonin

Oxygen homeostasis is essential to the optimal function of all cellular processes and especially for sustaining appropriate ATP levels. Ground state oxygen is an electron acceptor during numerous biochemical events and, considering its importance, it is anticipated that cells would have accommodated means to handle variations in intracellular oxygen tension. Rather than elevated oxygen levels, hypoxia is usually considered more consequential for cellular health and occurs more frequently than hyperoxia. The associated responses to depressed levels of oxygen are widely conserved and these adjustments can be made rapidly [131]. Hypoxia develops in cells because of an inadequate blood supply in the event of obstruction of vessels leading to the tissue, as a result of edema which constricts the capillaries or when neovasculogenesis cannot keep pace with rapidly proliferating cells such as in cancer. In these oxygen-deficient microdomains the cells may die and undergo necrosis, or they must adjust to survive [132]. As summarized above, the oxygen concentration within cells is recognized by HIFs [101,133] which are then upregulated.

**Figure 5:**
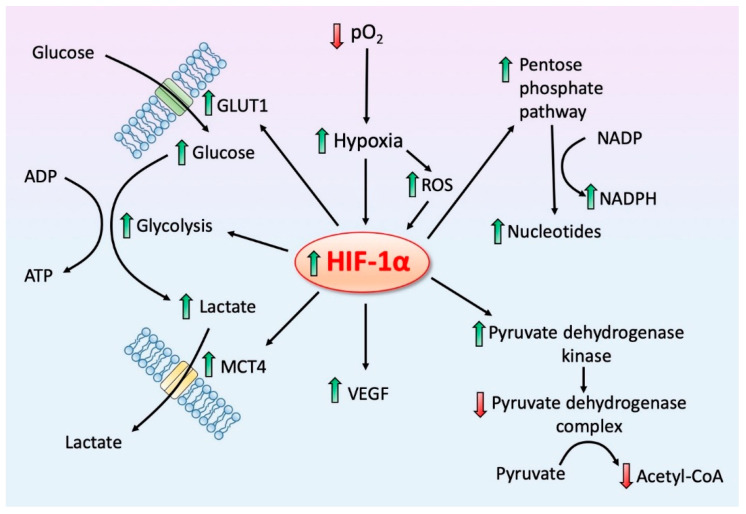
Hypoxia inducible factor-1 alpha (HIF-1α) is a central transcription agent in mediating Warburg-type metabolism within diseased cells. HIF-1α is part of an oxygen sensing system which becomes activated when the partial pressure (pO2) of intracellular oxygen becomes depressed, that is, when cells become hypoxic. A primary means by which HIF-1α promotes Warburg-type metabolism is by upregulating the mitochondrial enzyme pyruvate dehydrogenase kinase which, in turn, inhibits pyruvate dehydrogenase complex thereby reducing the conversion of pyruvate to acetyl coenzyme A in the mitochondria. The reduction of acetyl coenzyme A in the mitochondrial severely restricts the intramitochondrial synthesis of melatonin (see Figure 2). Further, HIF-1α promotes the pentose phosphate pathway (see Figure 4) and upregulates glycolysis by stimulating the glucose transporter (GLUT1), which accelerates the influx of glucose (the “sweet tooth” phenomenon), and promoting the efflux of lactate by stimulating the monocarboxylate transporter 4 (MCT4) which also stimulates neovasculogenesis by upregulating vascular endothelial growth factor (VEGF). Melatonin, by directly or indirectly inhibiting HIF-1α, reverses the Warburg effect and prevents the associated metabolic activities. Reactive oxygen species, which are produced more abundantly under hypoxic conditions, aid in the stabilization of HIF-1α.

In the context of the present report, one of the crucial actions of HIF-1α is its ability to stimulate the mitochondrial enzyme PDK which, in turn, downregulates PDC (Figure 5). PDC inhibition deprives the mitochondria of acetyl-CoA which is normally fed into the TCA cycle to provide reducing equivalents for OXPHOS. Additionally, however, PDC inhibition suppresses mitochondrial melatonin synthesis as well [125] (Figure 2). The loss of intracellular melatonin production may be an important aspect of the ability of cells to reprogram pyruvate processing and to progress to Warburg-type metabolism since melatonin is an endogenous inhibitor of HIF-1α [85,111,124].

The experimental evidence that melatonin, at both physiological and pharmacological concentrations, inhibits HIF-1α comes primarily from publications where melatonin/HIF-1α interactions were examined in cultured cancer cells. When prostate tumor cells were incubated with melatonin, under both normoxia and hypoxia, Park et al. [134] reported the reduced expression of HIF-1α but no downstream changes that would be expected to occur after HIF-1α inhibition were investigated. Under conditions of normal intracellular oxygen tension, HIF-1α is usually rapidly polyubiquitinated and then undergoes proteasome degradation [111]. During hypoxia, this does not occur and elevated HIF-1α is maintained leading to a rash of molecular events that enhance aerobic glycolysis and inhibit mitochondrial OXPHOS (Figure 5).

In similar studies, using cancer cells, melatonin reduced HIF-1α protein expression or destabilized this transcription factor [135,136,137,138,139]. This latter observation is consistent with what is known about the stabilizing effect of ROS and HIF-1α. In the presence of the antioxidant melatonin, the ROS concentration would be reduced thereby contributing to the destabilization of HIF-1α. These studies were most often directed at investigating the actions of melatonin on the neovascularization of tumors resulting from the modulation of vascular endothelial growth factor (VEGF). In cancer cells, VEGF is stimulated by HIF-1α and when these cells are exposed to melatonin, HIF-1α, VEGF and new vessel growth are hindered. Thus, whereas these reports repeatedly observed that HIF-1α is diminished by melatonin, none have examined the consequences of this treatment on the PDK/PDC/Warburg axis which can only be inferred from the results of studies showing disinhibition of PDC reduces cytosolic glycolysis and aids mitochondrial OXPHOS [140].

The observations of Blask and colleagues [84] as discussed above are convincing in suggesting that high endogenous nighttime melatonin levels downregulated the Warburg effect during the daily dark period. It is paradoxical, however, that when activated HIF-1α was measured in the tumors, relative to daytime levels they were elevated even in the presence of high circulating melatonin concentrations. This creates the conundrum that the aerobic glycolysis is suppressed even when HIF-1α is elevated at night which should enhance the Warburg effect. Moreover, it is contrary to other studies which report melatonin inhibits HIF-1α. The authors suggest two possible processes to explain this apparent discrepancy. They propose that since oxygen uptake may be suppressed by melatonin, it contributed to the intracellular hypoxia, a known promoter of HIF-1α activity [141,142]. In turn, the high HIF-1α would be expected to suppress mitochondrial PDC (via stimulation of PDK), which it did not do based on the glycolytic intermediates that were measured. How the failure of PDC inhibition was achieved remains unexplained.

As an alternative explanation, the authors suggested that HIF-1α may have been activated independently of intracellular hypoxia via the PI3K/AKT pathway resulting from the action of insulin growth factor-1 (IGF-1) since its receptors are upregulated in the xenografted tumors which would have promoted HIF-1α via the IGF-1/PI3K/AKT pathway [143]. Since neither potential explanation has been examined for validity, it is still a quandary as to how nighttime elevated HIF-1α did not prevent Warburg-type metabolism. This issue will only be resolved with additional investigation.

Although melatonin’s efficacy in reducing molecular damage and preserving cellular physiology during hypoxia caused by ischemia/reperfusion (I/R) injury has been extensively studied [144,145,146], the examination of the HIF-1α status as a result of melatonin treatment has been sparingly examined. I/R injury such as following the interruption of the blood supply to tissue or after organ transplantation causes a severe hypoxia with upregulation of HIF-1α [147,148]. Induced retinal ischemia in mice was followed by an elevated expression of HIF-1α and glial fibrillary acidic protein (GFAP). Melatonin, given shortly before ischemia induction, in addition to improving retinal cell survival inhibited HIF-1α stabilization and GFAP [149]. As a secondary observation in a rat neural I/R study, Kryl’skii et al. [150] noted that HIF-1α mRNA accumulated but was normalized along with a decrease in lactate levels when the animals were treated with melatonin.

Based on the findings from the cancer and I/R studies, both of which are pathological situations, HIF-1α is not rapidly degraded by the proteasome as in normal tissue. Melatonin treatment, however, increases the likelihood of ubiquitination and proteasomal destruction [111].

## 5. Melatonin Reprograms Glucose Metabolism: Converting Diseased Cells to a Healthier Phenotype

Table 1 is an incomplete list of pathological cell types, neoplastic and non-neoplastic, that exhibit aerobic glycolysis (the Warburg effect) along with references indicating that melatonin inhibits each of those pathologies. Here, we propose that melatonin’s ability to positively impact so many cell types stems from a common underlying mechanism, that is, its ability to re-route pyruvate metabolism from the cytosol to the mitochondria, thereby converting cells from their reliance on cytosolic aerobic glycolysis to mitochondrial OXPHOS. This also implies that each of the diseases mentioned is generally a “metabolic disease”.

The altered glucose metabolism, known as the Warburg effect, was initially discovered in cancer cells [222] but within the last two decades, it has also been found to occur in non-neoplastic, diseased cells as well. In the case of tumors, reversing the Warburg effect is considered a potential target for tumor therapy and several synthetic molecules have been developed for this purpose. The drugs are collectively known as glycolytics, the most thoroughly investigated of which is probably dichloroacetate (DCA) [223]. While DCA has cancer inhibiting actions, it has collateral toxicity at the level of the myelin sheaths of peripheral nerves [224]. Melatonin, to the authors knowledge, is the first endogenously produced molecule that has been shown to be capable of redirecting pyruvate metabolism away from the fermentation pathway and into the mitochondria for the support of the TCA cycle and OXPHOS [23,125]. Thus, the well-documented actions of melatonin on cancer progression [15,17,155,225] may be related to its ability to inhibit elevated cytosolic glycolysis of these cells [226]. Since this may be the case, it is also possible that melatonin’s efficiency in reducing the severity of other diseased cells that display this altered metabolism (see Table 1) may likewise be related to its ability to limit pyruvate conversion to lactate and to redirect it into the mitochondria, thereby normalizing the metabolism of this organelle and rendering the cells less-diseased/pathological [20]. Thus, the common denominator for melatonin to influence the progression of so many seemingly unrelated diseases may be a consequence of its action in determining the route of pyruvate metabolism (Figure 2).

Since endogenous melatonin levels increase in the blood on a nightly basis in most individuals, and given that melatonin readily presumably enters all cells [80,81,83] as well as their mitochondria [64,81,227] with equal facility, it would be expected that diseased cells (both neoplastic and non-neoplastic) may be metabolically different at night than during the day. At least for xenografted human breast cancer cells grown in animals, this has been shown [84]. Thus, these tumors clearly exhibit the Warburg effect during the day, characterized by the massive synthesis and release of lactate; at night, they do not, that is, they switch their metabolism away from pyruvate fermentation. This redirected metabolism is unequivocally related to the nocturnal rise in circulating melatonin since eliminating the large nighttime increase of pineal-derived melatonin by the exposure of the animals to light at night clearly prevents the reversal of pyruvate metabolism. On the basis of these findings, we reasoned that cancer cells, are, in fact, only of the cancer phenotype about half the time (during the day) [85,125]. This can now also be extrapolated to other diseases that opt for a Warburg-type metabolism. Hence, the non-neoplastic diseases summarized in Table 1 (and there are others) may also be of a pathogenic phenotype only 50% of the time while at night they may function with a healthier phenotype. As with cancer cells, this day: night difference has not been observed in any disease that is not neoplastic. The reason for this failure to identify this switch is, however, obvious. The metabolism of all diseased tissues is routinely examined during the day or in cultured cells with the assumption that the same type of metabolism occurs 24/7, which may not be the case. It is obvious that, to get an accurate estimate of the metabolism of diseased tissue, it must be evaluated during both the day and at night; the latter tissues must be collected under circumstances where the nighttime rise of blood melatonin levels is not compromised. Furthermore, for such studies, it would be futile to examine cells growing in culture since they are never exposed to the circadian melatonin rhythm.

In diseased cells, especially, the pineal-derived melatonin rhythm must be considered in relation to the intramitochondrial synthesis of this critical molecule. In healthy cells, mitochondria produce melatonin both during the day and at night, so it is always available to inhibit HIF-1α/PDK axis which allows for the upregulation of PDC and the conversion of pyruvate to acetyl-CoA, the necessary co-factor/co-substrate for melatonin synthesis (Figure 2). In contrast, in diseased cell mitochondria, melatonin synthesis does not occur because of some not yet identified metabolic change, e.g., drop in PO_2_, which upregulates HIF-1α, thereby shutting down mitochondrial acetyl-CoA production and melatonin formation. At night, pineal-derived melatonin in the blood enters diseased cells to inhibit HIF-1α, allowing them to switch back to the usual type of metabolism, thus making them more normal, healthier at night [64,85,228].

Pineal melatonin synthesis, however, usually diminishes with increasing age in both animals and humans [229,230,231,232]. This also presumably occurs at least to some degree in the mitochondria [233]. As a result, in elderly subjects, all cells exist in a relatively less-healthy state and the cells that become diseased (both neoplastic and non-neoplastic) persist as such both during the day and at night, allowing them to progress in their pathogenic condition at a greater frequency and a more rapid rate. This theoretically could explain the increased vulnerability of the older population to the elevated occurrence and more rapid development of diseases.

To restore the nighttime rise in blood levels of melatonin, taking it as a supplemental, especially by the elderly, may be beneficial in slowing the progress of age-related diseases by ensuring that the mitochondria are engaged in OXPHOS at least a portion of every 24-h period, as occurs in younger individuals who still experience a nocturnal increase in endogenous melatonin. Administration of melatonin is followed by an elevation in blood melatonin concentrations [234] from which it is readily taken up. Melatonin can be administered orally, sublingually, intranasally rectally as a suppository, intravenously, and as a skin patch containing absorbable melatonin. In addition to being available in conventional powdered form, it has been incorporated into nano-formulations, which may increase its bioavailability, especially at the mitochondrial level [235].

The safety of melatonin has been thoroughly examined [236,237]. It is not a foreign substance for humans (or any vertebrate species) since it is endogenously produced. Even when given at extremely high doses, its side-effects are minimal [(occasional headache or sleepiness) and no lethal dose has yet been established, despite the administration of massive amounts. Extremely rare idiosyncratic reactions have been observed [238,239].

The potential of melatonin in reducing the likelihood of developing or limiting the severity of disease in humans has long been speculated and there is evidence supporting its utility in deferring age-related diseases. Moreover, the well-being of individuals who routinely use melatonin as a supplement is reported to be improved [221].

Serious diseases of the frail and elderly are obviously highly debilitating, often involve the entire family group, and are very expensive to treat. Some chronic diseases in the older population cost the healthcare system many millions of dollars annually. If melatonin can successfully defer the onset of these conditions, which it may do, in addition to improving the well-being of these individuals, it would also be fiscally beneficial. While melatonin’s use by the older population may be highly significant, young individuals diagnosed with the diseases summarized in Table 1 may also benefit from at least short-term melatonin use; this is especially relevant for individuals who routinely experience disruptive light: dark cycles, for example, shift workers. Considering the potentially high efficacy of melatonin as a disease-deferring molecule, controlled clinical trials using it such as those currently planned for COVID-19 infections are certainly warranted and badly needed. Unfortunately, as an inexpensive and non-patentable molecule, there is little enthusiasm for these studies from the pharmaceutical industry.

## 6. Melatonin: A Molecular Peacekeeper during Troubled Metabolic Times

Massive amounts of financial and investigative effort have been spent in attempts to unravel the underpinnings of the mechanisms which aid cancer cell biomass to rapidly expand and become resistant to radio- and chemo-therapies. Intensive investigations have potentially unraveled this Gordian knot but the entanglement has only stubbornly revealed the underlying molecular events related to observed accelerated cell proliferation and opposition to cancer therapies; in many cases, what is known about details of the subcellular transformation contributing to the changes seemingly remain sketchy and vary among cancer types.

One feature that is common to many solid tumors is their adjustment of glucose metabolism such that its metabolite, pyruvate. Rather than being transported into the mitochondria to support ATP production via OXPHOS it is retained in the cytosol where it is converted to lactate. The abandonment of OXPHOS in lieu of cytosolic pyruvate metabolism is commonly referred to as aerobic glycolysis or the Warburg effect. This transformation is associated with the rapid albeit inefficient, ATP synthesis as a result of accelerated glycolysis as well as “opening the flood gate” for the pentose phosphate pathway (Figure 4). These changes collectively provide the enhanced energy levels and the augmented structural molecules required to support the intensive cellular proliferation cancer cells often display. However, the Warburg effect has advantages beyond its value in supporting molecular synthetic activity such as often making the tumor cells resistant to conventional therapies, etc. The Warburg effect is a devious metabolic scheme to enhance the cancer and protect it from negative influences.

In addition to tumors, many other non-cancer tissues likewise favor Warburg metabolism in lieu of mitochondrial OXPHOS. This energy-generating shift is most often observed in pathological situations. As with cancer, the data overwhelmingly indicates that suppressing Warburg-type metabolism alleviates the diseased condition in which this metabolic phenotype is found.

As already mentioned earlier, molecules/drugs which interrupt the Warburg effect and allow cells to select mitochondrial OXPHOS as their energy source are referred to as glycolytics. These synthetic molecules, since they reverse a pathological type of metabolism, would be expected to be beneficial and, in general, they are. The downside is that since they are foreign to the organism, they may have side-effects.

Melatonin functions as a glycolytic since it also inhibits the Warburg effect. Melatonin may also have advantages over the synthetic glycolytics since it is an endogenously synthesized molecule in every organism including in the human. Hence, it is assumed that, at least at the physiological level, it is not harmful. All organisms live their life with this molecule and it is the loss of melatonin that often compromises organ and organismal physiology. It is commonplace, however, that it is essentially always given in amounts that cause blood levels to exceed those that are normally measured and identified as being physiological, that is, melatonin application typically leads to pharmacological concentrations in the blood. However, given that intracellular levels of melatonin in some cellular organelles seemingly greatly exceed the concentrations measured in the blood, the amounts of supplemental melatonin normally taken may not be pharmacological for every compartment.

## Figures and Tables

**Table 1:** A partial list of cell types that can experience cytosolic aerobic glycolysis. In most cases, the Warburg metabolism is considered to be associated with diseased cells; while this is almost universally valid, it is not absolute. Solid cancers, a sampling of which is listed here, often adopt Warburg-type metabolism in lieu of mitochondrial oxidative phosphorylation for ATP production. In some cells, however, both routes of pyruvate metabolism and ATP production are preserved. The table also includes an incomplete list of non-tumor, but diseased, cells which employ aerobic glycolysis. Only a single reference is cited in support of each category. In some cases, there are many more publications that document the occurrence of Warburg-type metabolism. Experimentally, melatonin has been shown to inhibit each of the pathological cell types listed (both cancer and non-cancer). Here, we propose that melatonin’s ability to reprogram cytosolic aerobic glycolysis to mitochondrial oxidative phosphorylation, at least in part, may be a unifying mechanism by which melatonin negatively impacts diverse pathological cells.

Cell Types that Display Warburg Effect	References Reporting Warburg Metabolism	References Indicating Melatonin Inhibits these Cell Types
	**Cancer**	
Ewing sarcoma	Yeung et al. [151]	Sanchez-Sanchez et al. [124]
Osteosarcoma	Shen et al. [152]	Lu et al. [153]
Breast	Tailor et al. [154]	Hill et al. [155]
Hepatocellular carcinoma	Li et al. [156]	Elmahallway et al. [157]
Glioblastoma	Du et al. [158]	Moretti et al. [159]
Thyroid cancer	Yang et al. [160]	Liao et al. [161]
Non-small cell lung	De Rosa et al. [162]	Ma et al. [163]
Prostate	Schoepke et al. [164]	De Almeida Chuffa et al. [165]
Ovarian	Freidus et al. [166]	Bu et al. [167]
Colorectal	Fu et al. [168]	Gil-Martin et al. [169]
Pancreatic	Annas et al. [170]	Tamtaji et al. [171]
Cervical	Zhang et al. [172]	Wang et al. [173]
Stomach	Liu et al. [174]	Liu et al. [175]
Melanoma (cutaneous)	Kumar et al. [176]	Alvarez-Artime et al. [177]
Myeloproliferative	Baumeister et al. [178]	Shafabakhseh et al. [179]
Bladder	Alfonso et al. [180]	Chen et al. [181]
Endometrial	Salama et al. [182]	Gu et al. [183]
Renal Cell Carcinoma	Lessi et al. [184]	Wen et al. [185]
	**Non-Cancer Diseases**	
Multiple sclerosis	Kornberg et al. [186]	Lopez-Gonzalez et al. [187]
Alzheimer disease	Altante et al. [188]	Rosales-Corral et al. [189]
Huntington disease	Damiano et al. [190]	Wongprayoon and Govitrapons et al. [191]
Amyotrophic lateral sclerosis	Vallee et al. [192]	Luo et al. [193]
Parkinson disease	Tang et al. [194]	Chen et al. [195]
Polycystic kidney disease	Podrini et al. [196]	Millet-Boureima et al. [197]
Diabetic kidney disease	Morita and Kawaski et al. [198]	Promsan and Lungkaphin et al. [199]
Glaucoma	Del Valle et al. [200]	Yu et al. [201]
Fibrosis	Tian et al. [202]	Jiang et al. [203]
Pulmonary hypertension	Cottrill and Chan [204]	MacLean [205]
Myocbacterium tuberculosis	Krawczyk et al. [206]	Wiid et al. [207]
Septic shock	Ji et al. [208]	Colunga-Biancatelli et al. [209]
Atherosclerosis	Ma et al. [210]	Sezgin et al. [211]
Human papilloma virus	Martinez-Ramirez et al. [212]	Ma et al. [213]
Herpes simplex	Di Sotto et al. [214]	Nunez-Oda and Pereira -Rdo [215]
SARS-CoV-2	Icard et al. [216]	Ramlall et al. [217]
Viral infected hepatocytes	Tarasenko et al. [218]	Crespo et al. [219]
HIV/AIDS	Dagenais-Lussier et al. [220]	Lissoni et al. [221]

## Data Availability

No data included.
